# Principles for creating a single authoritative list of the world’s species

**DOI:** 10.1371/journal.pbio.3000736

**Published:** 2020-07-07

**Authors:** Stephen T. Garnett, Les Christidis, Stijn Conix, Mark J. Costello, Frank E. Zachos, Olaf S. Bánki, Yiming Bao, Saroj K. Barik, John S. Buckeridge, Donald Hobern, Aaron Lien, Narelle Montgomery, Svetlana Nikolaeva, Richard L. Pyle, Scott A. Thomson, Peter Paul van Dijk, Anthony Whalen, Zhi-Qiang Zhang, Kevin R. Thiele

**Affiliations:** 1 Research Institute for the Environment and Livelihoods, Charles Darwin University, Darwin, Australia; 2 Southern Cross University, Coffs Harbour, Australia; 3 Centre for Logic and Philosophy of Science, KU Leuven, Leuven, Belgium; 4 School of Environment, University of Auckland, Auckland, New Zealand; 5 Faculty of Biosciences and Aquaculture, Nord University, Bodø, Norway; 6 Natural History Museum Vienna, Vienna, Austria; 7 Department of Evolutionary Biology, University of Vienna, Vienna, Austria; 8 Department of Genetics, University of the Free State, Bloemfontein, South Africa; 9 Species 2000, Naturalis, Leiden, the Netherlands; 10 National Genomics Data Center, Beijing Institute of Genomics (China National Center for Bioinformation), Chinese Academy of Sciences, Beijing, China; 11 CSIR-National Botanical Research Institute, Rana Pratap Marg, Lucknow, India; 12 Earth & Oceanic Systems Group, RMIT, Melbourne, Australia; 13 Museums Victoria, Carlton, Australia; 14 Species 2000, Canberra, Australia; 15 School of Natural Resources and the Environment, University of Arizona, Arizona, United States of America; 16 Arizona Institutes for Resilience, University of Arizona, Arizona, United States of America; 17 Department of Agriculture, Water and the Environment, Canberra, Australia; 18 Sessional Committee, Scientific Council, Convention on Migratory Species, Bonn, Germany; 19 Department of Earth Sciences, The Natural History Museum, London, United Kingdom; 20 Kazan Federal University, Kazan, Russia; 21 B. P. Bishop Museum, Honolulu, Hawai’i, United States of America; 22 Chelonian Research Institute, Oviedo, Florida, United States of America; 23 Global Wildlife Conservation, Austin, Texas, United States of America; 24 Manaaki Whenua-Landcare Research and School of Biological Sciences, The University of Auckland, Auckland, New Zealand; 25 Taxonomy Australia, Australian Academy of Science, Canberra, Australia

## Abstract

Lists of species underpin many fields of human endeavour, but there are currently no universally accepted principles for deciding which biological species should be accepted when there are alternative taxonomic treatments (and, by extension, which scientific names should be applied to those species). As improvements in information technology make it easier to communicate, access, and aggregate biodiversity information, there is a need for a framework that helps taxonomists and the users of taxonomy decide which taxa and names should be used by society whilst continuing to encourage taxonomic research that leads to new species discoveries, new knowledge of species relationships, and the refinement of existing species concepts. Here, we present 10 principles that can underpin such a governance framework, namely (i) the species list must be based on science and free from nontaxonomic considerations and interference, (ii) governance of the species list must aim for community support and use, (iii) all decisions about list composition must be transparent, (iv) the governance of validated lists of species is separate from the governance of the names of taxa, (v) governance of lists of accepted species must not constrain academic freedom, (vi) the set of criteria considered sufficient to recognise species boundaries may appropriately vary between different taxonomic groups but should be consistent when possible, (vii) a global list must balance conflicting needs for currency and stability by having archived versions, (viii) contributors need appropriate recognition, (ix) list content should be traceable, and (x) a global listing process needs both to encompass global diversity and to accommodate local knowledge of that diversity. We conclude by outlining issues that must be resolved if such a system of taxonomic list governance and a unified list of accepted scientific names generated are to be universally adopted.

## Abstract 2

Currently there are no rules on compiling lists of accepted names of species, meaning that many people use a variety of competing lists, which results in confusion and inefficiencies. This Perspective article provides principles for developing global lists of species that are needed if such lists are to be accepted by both taxonomists and users of taxonomy.

## Introduction

Lists of species matter. Whether a species (or any other taxon) is included on a list may affect conservation (e.g., by affecting investment in threatened species), trade (e.g., through the Convention on International Trade in Endangered Species of Wild Fauna and Flora [CITES] or listing as an invasive pest), development (through national threatened species legislation), local livelihoods (through species-specific conservation programs, ecotourism, etc.), or evolutionary and ecological research (e.g., in diversification analyses and macroecological studies). In the face of an unfolding global extinction crisis, global lists of accepted species are foundational to managing biodiversity in an era of accelerating global change.

In view of the importance and wide usage of lists of accepted species, there has been a push to create a unified, authoritative, comprehensive, and global list that is accepted by both the scientific community and key users [1–3]. The current lack of such a list has 4 main negative consequences. First, it means that some of the world’s named species are effectively invisible to those who lack the resources to access or navigate specialist taxonomic literature. Second, the lack of a unified list means that scientific contributions towards improving the quality of taxonomic and nomenclatural knowledge are scattered and can often be missed. Third, it forces other users, who may lack relevant taxonomic expertise, to choose between competing lists and taxonomic treatments of groups even though they rarely understand the rationale why competing lists exist and are confused by the differences between alternate taxonomic concepts and classifications. Fourth, users may follow regional or other species identification guides or lists without realising that they are outdated and should be checked against authoritative sources [4].

One of the difficulties of creating a unified global list of accepted species is that the circumscription of a taxon is not a fully objective decision. It includes a judgement as to whether a set of specimens or populations is best regarded as one or more than one species [5–9]. Different taxonomists may legitimately judge the limits of taxa differently even when using the same data, and there are many alternative sources of data and methods for delimiting taxa (e.g., using molecular or morphological phylogenetics, morphometrics, or ecological or physiological traits).

All lists of accepted species are founded on the efforts of taxonomists describing and classifying new taxa, revising existing taxa, and, often using new data and methods, relegating to history taxa that are shown to be poorly justified or defined. Taxonomic research often involves reviewing the merits of existing taxa [10], a process that includes reviews of larger taxonomic groupings such as genera, families, orders, classes, phyla, and kingdoms at global scales. At high taxonomic levels, for all but the least species-rich groups, it is usual for consolidated global lists to combine the work of multiple taxonomists.

Traditionally, consolidated species lists consisted of formal publications that were produced at irregular intervals and not updated for decades. Increasingly, they are actively curated online with more frequent updates [11,12]. At the highest level, various organisations aggregate lists across multiple groups. Such multigroup lists are relatively common at the national and infranational level, at least for plants and vertebrates, particularly where listing has legal standing.

**Fig 1:**
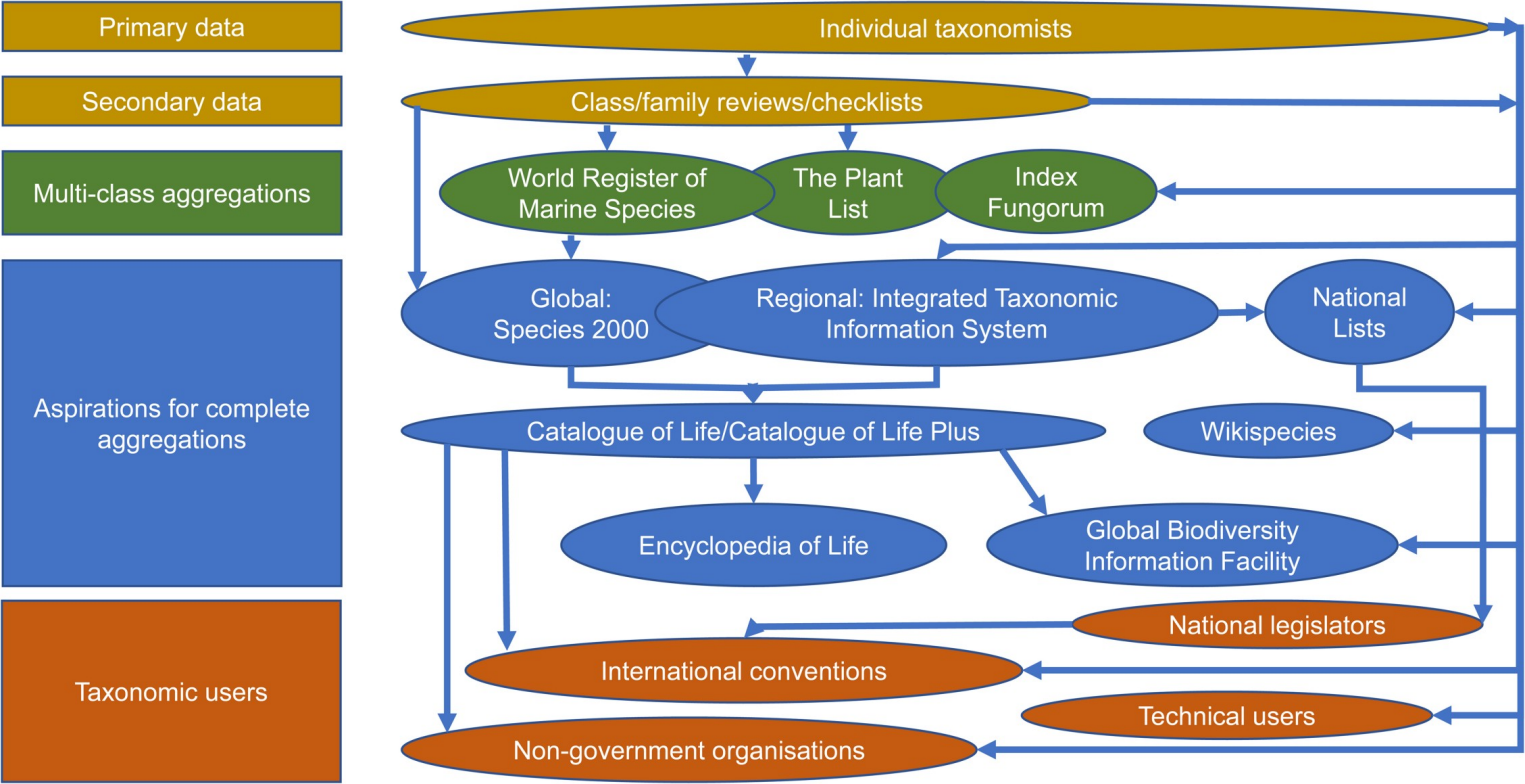
Process by which taxonomic information is currently assembled into global lists. At just about every stage, the taxonomic decisions of individual taxonomists can influence lists directly, as well as through the various levels of aggregation.

Two decades ago, the late Frank Bisby championed a single authoritative list of all species on Earth [13,14]. A partnership between the International Council for Science (ICSU: Committee on Data for Science and Technology), the International Union of Biological Sciences (IUBS), and the International Union of Microbiological Societies in the early 1990s resulted in the launch of the Catalogue of Life (CoL), hosted by Species 2000. The CoL is currently fairly complete for major taxonomic groups, with gaps being narrowed rapidly through greater participation by the taxonomic community. It brings together 173 individual global lists, referred to as Global Species Databases (GSDs), covering much biodiversity [15]. Numerous organisations use the CoL as their taxonomic backbone to resolve the correct spellings, classification and synonymies of species names, including the Global Biodiversity Information Facility (GBIF) and the Encyclopedia of Life (EoL), as well as international biodiversity policy initiatives such as the Convention on Biological Diversity (CBD), the United Nations Environment Program, and the European Environment Agency. The World Register of Marine Species (WoRMS) provides numerous global species lists to the CoL and independently provides species name resolution services to GBIF, EoL, and the Ocean Biogeographic Information System [11,12]. Thus, whilst there is already a high degree of collaboration among aggregators, with much sharing of lists (Fig 1), all the aggregators remain distinct entities. This results in differences among the lists they propagate, not least because they are updated at different rates. Added to this are the complications that arise when taxonomic groups have multiple authoritative lists. As an example, there are currently at least 4 global lists of birds, each differing in their taxonomic approach and each having their own adherents [1], causing confusion and inefficiencies among list users and diverting resources away from users’ core purposes.

Consolidation requires decisions as to which taxa to accept and which to reject. In many areas of science beyond taxonomy, organisations have made decisions, even controversial ones, on issues that transcend national borders. To do so, they have invoked governance principles to adjudicate decisions even in the face of continued debate. As an example, the decision in 2006 by the General Assembly of the International Astronomical Union that Pluto was not a full planet proved highly controversial and severely tested the governance of the organisation [16]. Similarly, the process of deciding whether to recognise the ‘Anthropocene’ as a new geological era is being debated by the International Union of Geological Sciences [17]. Beyond science, many international agreements often have strong dispute resolution mechanisms, especially when there are power discrepancies between parties [18]. However, when there are differences in opinion about species validity, there are no formal rules for dispute resolution or frameworks within which the differences can be managed. Whilst progress towards reconciliation between different taxonomic lists is currently underway through the CoL Plus endeavour and the Alliance for Biodiversity Knowledge [19], opposing views are deeply entrenched in some groups, and reconciling them will be no trivial task.

Nevertheless, much can be learnt from the communities of practice that already exist and regularly make difficult taxonomic decisions to create aggregated lists at a regional level or for major taxonomic groups. To achieve global acceptance, a unified global list of species will only be adopted if the full range of stakeholders in lists find it advantageous to use. Amongst these stakeholders will be not only list users such as naturalists, ecologists, the commercial sector, and government agencies but also the community of taxonomists. Fully engaging with this community will be essential if an authoritative, widely adopted unified global list of accepted species covering all forms of life is to be created.

Here, we set out a series of principles (Box 1) that we believe will support existing efforts to create a global list with the legitimacy and institutional authority needed to facilitate adoption as a global standard by governments and other national and international institutions. Whilst we envision the final product to consist of a single, global, unified list of species, we expect such a master list to emerge as an aggregate of multiple well-defined lists focused on specific taxonomic groups. The principles we describe apply to both the constituent lists and the resultant aggregated unified list.

**Box 1. Ten principles proposed as the basis of global species lists**

1. The species list must be based on science and free from nontaxonomic considerations and interference.
2. Governance of the species list must aim for community support and use.
3. All decisions about list composition must be transparent.
4. The governance of validated lists of species is separate from the governance of the naming of species.
5. Governance of lists of accepted species must not strain academic freedom.
6. The set of criteria considered sufficient to recognise species boundaries may appropriately vary between different taxonomic groups but should be consistent when possible.
7. A global list must balance conflicting needs for currency and stability by having archived versions.
8. Contributors need appropriate recognition.
9. List content should be traceable.
10. A global listing process needs both to encompass global diversity and to accommodate local knowledge of that diversity.

## Principles

### 1. The species list must be based on science and free from nontaxonomic considerations and interference

Taxonomy, including the creation and maintenance of lists of accepted species, is a foundational science that has a wide range of important implications. Whilst development of the governance processes for aggregating lists may involve the users of lists, decisions about the creation of a unified global list and its components must be independent of political, economic, or other nontaxonomic considerations if they are to have scientific legitimacy and be widely adopted. As an example, a taxonomic list that has been crafted or optimised for conservation purposes may lack legitimacy for other purposes. This principle also extends to how the governance of a global list is funded: the funding body must neither directly nor indirectly interfere with the governance process. Hence, funding for the creation and maintenance of a global list needs to be separated transparently in the governance process from any decisions related to the taxonomy itself.

### 2. Governance of the species list must aim for community support and use

The governance processes underpinning an authoritative list must be recognised and supported by both taxonomists and the users of taxonomy. Currently many users of taxonomy have no means of having their views heard in the construction of lists. As the easiest response to a lack of power is to refuse to participate [20], there needs to be active engagement by a wide range of potential users if a unified global list is to be globally supported. To ensure this, the needs of all interested parties should be considered when designing and implementing the governance system. A global authoritative list of species should support different derived products tailored for different users, such as the scientific community, nature management authorities, policymakers, and the broader public with an interest in nature. In addition, the governance processes should be validated by important international organisations. This should include, at the highest level, international institutions such as the IUBS and International Science Council (ISC) so that users have confidence that the processes have the integrity and authority of the highest level of international governance available to science.

### 3. All decisions about list composition must be transparent

The legitimacy of listing will rest partly on the transparency of its decision-making processes. Like many scientific bodies, the process for developing an agreed list needs to balance expertise and inclusion. One useful model may be wiki technology and open source software communities, in which individuals earn greater influence by contributing in an appropriate and cooperative manner, as happens with Wikispecies and WoRMS [11]. For any aggregation of taxonomies, there must be rules about who decides which taxonomies are accepted if there are alternative treatments (often, there are not), how such decisions are reached, and how debates are resolved. This will require documenting the opinions of recognised experts, any differences of opinion, and the processes used to reconcile alternative views. To be fully transparent, the list itself must be fully open access, archived, and with a conventional citation that indicates who was responsible for its editing and where it was published, as exemplified by the CoL [21].

### 4. The governance of validated lists of species is separate from the governance of the names of taxa

In discussing list governance, it is important to distinguish the development of a list of accepted species from taxonomic nomenclature. Careful governance of names is a necessary underpinning of the governance of a unified list of taxa. The nomenclatural rules that govern the naming of organisms (the International Codes of Nomenclature) have been developed, managed, and finessed for over a century and so far have proven to be resilient, flexible, and fit for purpose to ensure the orderly naming of taxa. The Codes (e.g., International Code of Nomenclature of Prokaryotes, International Code of Zoological Nomenclature, International Code of Nomenclature for algae, fungi, and plants) and associated resources (e.g., ZooBank, Index Fungorum, MycoBank, International Plant Names Index) continue to be actively maintained by globally recognised governance structures. These well-developed governance arrangements for nomenclature can serve as models for what can be achieved for the governance of a unified list. However, these are separate activities and must be kept separate for the integrity of both. Although the governance of nomenclature should not interfere with the governance of a unified and authoritative reference list of species (and vice versa), lists in both domains should be technically compatible and, whenever possible, tightly integrated.

### 5. Governance of lists of accepted species must not constrain academic freedom

Like all other sciences, taxonomy requires freedom of research and expression to remain vital and make progress. Concerns have been expressed that governance of a single unified list of accepted species may reduce the academic freedom of taxonomists (e.g., [22]). In our context, the governance process must aim to provide quality assurance, rigor, standardisation, and transparency and ensure that multiple views are considered when these are available. Within this framework, the freedom of taxonomists must remain unconstrained because the governance mechanisms do not apply to taxonomic research, but rather, to the way in which the results of taxonomic research are aggregated. In fact, it is anticipated that transparent governance of a unified global list will enhance the scientific reputation of taxonomy by showcasing the strengths and merits of both the decision-making process and the underlying taxonomic research on which the list is based.

### 6. The set of criteria considered sufficient to recognise species boundaries may appropriately vary between different taxonomic groups but should be consistent when possible

There is no universally accepted definition of a species. Whilst this may be considered a serious problem for taxonomy given the wide range of definitions that can legitimately be applied [23], in practice, it has not precluded the discovery and delimitation of taxa throughout the tree of life. For sound reasons, taxonomists working with different groups of organisms at times adopt different species concepts. However, consistency, utility, and acceptability of a global species list will be maximised if common approaches to delimiting species are adopted as widely as possible, at least within taxonomic groups. At times, governance rules for a global species list will need processes for deciding appropriate approaches to recognising species and clear standards for justifying these decisions. Traditionally, such decisions have been made by following the opinion of responsible experts. However, aggregators need agreed-upon guidance on how to choose when the views of equally responsible experts diverge.

### 7. A global list must balance conflicting needs for currency and stability by having archived versions

Taxonomy is a dynamic process, and debate about taxonomic circumscriptions and relationships is an irreducible outcome of taxonomic research. As with other scientific hypotheses, taxonomies attain legitimacy (or are overturned) through constant testing, with new, sometimes competing, hypotheses proposed, tested against new evidence, discussed, and ultimately adopted, modified, or rejected. Some users may be institutionally unable to accommodate rapid change and thus may prefer stability over currency. As an example, transaction and opportunity costs of changing national legislation and international conventions to accommodate taxonomic progress can be high if they result in laws, schedules, and agreements lagging taxonomic currency by years or decades [24]. One transparent process that balances the needs of all users is to ensure that dynamic sections of the list are published regularly as versioned, permanently accessible archives. Legislators and others whose need for stability is greater than their need for currency may choose to reference a designated, date-stamped, ‘frozen’ version of the list or of a single taxon as represented in the list. They must be able to access that version for as long as needed, even while the current list is updated. If a unified list enables the history of changes in taxa and names to be traced between versions, the onus is then on legislators, not taxonomists, to use a version that is acceptable and to update legislation at a rate commensurate with need.

### 8. Contributors need appropriate recognition

Taxonomy is a vocation for many taxonomists, an activity pursued well beyond the hours of normal employment or, in many cases, not remunerated at all—e.g., amateurs were responsible for 62% of the new terrestrial and freshwater multicellular species described in Europe between 1997 and 2008 [25], and many taxonomists remain active in research and publication long after retirement. Any global list will draw heavily on such dedication. Whilst many taxonomists derive intrinsic pleasure from their work, the most common extrinsic reward is through recognition by peers (and sometimes the broader public) through publications, particularly the description of new taxa. However, list preparation and annotation require effort, which should also be recognised, so any global listing system will need a mechanism for citation and public recognition of the taxonomists and organisations whose skills have been drawn on most heavily to give the list legitimacy. The use of a formal citation of a list that includes editors’ names, as conducted by the CoL [21], enhances its authoritativeness and ensures that users immediately know who was responsible for its quality. It also gives formal permanent credit to the people who created it. An attribution mechanism may track fine-scale microcontributions (as in Wikispecies), with periodic publication of parts or revised sections of the list in online open-access journals. Open citation also enhances transparency. Just as the names of authors who erect or revise species names are acknowledged, so too should be the names of those who compile and validate the taxonomic list.

### 9. List content should be traceable

The global species list should include full citations of the literature that originally established the taxa and their names, as well as of the taxonomic treatments used as the basis of the accepted status (classification and synonymy) for each taxon, to ensure explicit reference to both the underlying scientific nomenclature and the basis for the specific associated taxonomic concept.

### 10. A global listing process needs both to encompass global diversity and to accommodate local knowledge of that diversity

Given that biodiversity is global, that taxa are not constrained by national boundaries, and that there is a strong need for efficiencies of scale in any listing process, we believe that the appropriate scale for a unified list of accepted species and other taxa is global. However, national and infranational lists are also important, particularly when taxonomies are embedded in national and infranational legislation. Further, much taxonomic expertise is local rather than global; many highly competent taxonomists, for sensible reasons, have deep knowledge of taxa in their region but lack a global perspective on their taxonomic group. Global lists are only likely to be adopted locally if they are assembled with reference to local taxonomic knowledge.

## A pathway to adoption

Current systems provide a solid foundation for the creation and management of global lists, with the CoL Consortium having a version that is progressing rapidly. However, neither the CoL nor any of the other aggregative lists have been adopted universally by users of taxonomic research. There are likely to be many reasons for this. Some are practical. The work involved in changing a taxonomy at a local or national level may be greater than the resources available to do so, and the savings from adopting a global list may not be apparent at a local level. More significantly, no existing list mechanism has established clear buy-in from either the broad taxonomic community or the broad stakeholder community. We believe that achieving such buy-in will require adoption of the principles listed above.

**Fig 2:**
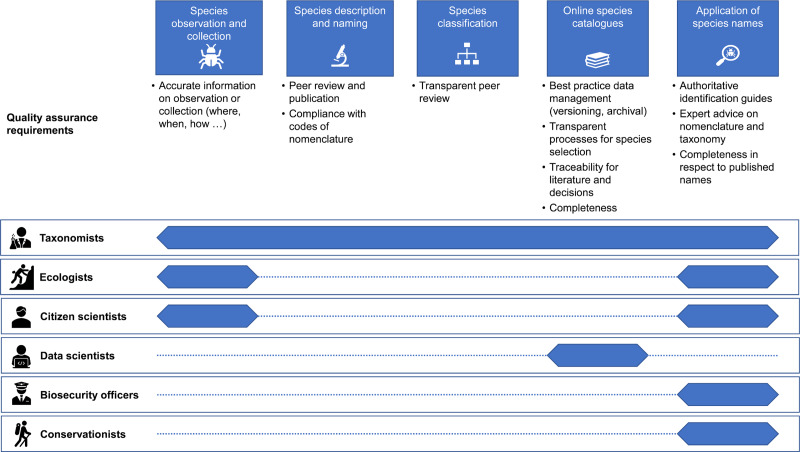
Steps in the process of global list aggregation highlighting current gaps.

For reasons outlined above, we believe that a key step in establishing a mechanism for creating and maintaining a unified global list that will maximise engagement from both the taxonomy sector and the stakeholder and user community is to establish a governance mechanism that operates at a high level of global science (Fig 2). No such mechanism currently exists that combines management of a unified list by a very broad-based community of taxonomists with an agreed endorsement and governance mechanism provided by a respected global body. Existing mechanisms have prioritised data management and the listing process over review and oversight structures.

We believe that a pathway to adoption of a successful global listing mechanism must include the following steps:

1. Refinement and agreement of a set of principles, as outlined above;
2. Establishment, by a representative global peak body such as the IUBS, of a governance mechanism based on the agreed principles;
3. Establishment, in collaboration with existing thematic groups and global and national mechanisms for creating taxonomic lists, of a framework, standards, and workplan for integrating existing competing lists, filling gaps in which no recent lists exist, and maintaining and managing the unified global list once created; and
4. Endorsement of the agreed mechanisms by key users of lists such as CITES, the International Union for Conservation of Nature (IUCN), the CBD, the Intergovernmental Platform for Biodiversity and Ecosystem Services (IPBES), the Convention on Migratory Species (CMS), and equivalent national bodies.

A mechanism such as this, based on transparent and agreed principles, is most likely to achieve successful establishment and adoption of a global list of accepted species. The global digital revolution provides both the need and many of the technological mechanisms to enable this pathway and its adoption. However, the governance framework must first be agreed by both taxonomists and key users of taxonomy to pre-empt many of the foreseeable impediments to adoption. To this end, the IUBS is supporting a pilot project on the governance of a unified global species list from 2020–2022 aiming to elaborate on the principles of governance of a global taxonomic list as provided above.

If realised, an authoritative global species list will be a remarkable achievement, both for global science and as an important part of a package of measures to respond to global challenges including the unfolding extinction crisis. A global taxonomic list will transcend borders, individual preferences, politics, and history. Development and adoption of such a system will be the work of decades, an accretion of small actions to improve and refine existing systems, rather than a revolution. Its consequences, however, may be revolutionary.

## Abbreviations

CBD: Convention on Biological Diversity
CDU: Charles Darwin University
CITES: Convention on International Trade in Endangered Species of Wild Fauna and Flora
CMS: Convention on Migratory Species
CoL: Catalogue of Life
EoL: Encyclopedia of Life
GBIF: Global Biodiversity Information Facility
GSD: Global Species Database
ICSU: International Council for Science
IPBES: Intergovernmental Platform for Biodiversity and Ecosystem Services
ISC: International Science Council
IUBS: International Union of Biological Sciences
IUCN: International Union for Conservation of Nature
WoRMS: World Register of Marine Species
